# Porous metal block based on topology optimization to treat distal femoral bone defect in total knee revision

**DOI:** 10.1007/s10237-023-01692-8

**Published:** 2023-01-25

**Authors:** Jiangbo Zhang, Aobo Zhang, Qing Han, Yang Liu, Hao Chen, Mingyue Ma, Yongyue Li, Bingpeng Chen, Jincheng Wang

**Affiliations:** 1 Department of Orthopedics, The Second Hospital of Jilin University, Changchun, 130041 China; 2 Department of Breast Surgery, The First Hospital of Jilin University, Changchun, 130021 China

**Keywords:** Finite element analysis, Total knee revision, Topology optimization, Porous design, Metal block augmentation

## Abstract

Metal block augmentations are common solutions in treating bone defects of total knee revision. However, the stress shielding and poor osteointegration resulted from metal block application could not be neglected in bone defects restoration. In this study, a novel porous metal block was designed with topology optimization to improve biomechanical performance. The biomechanical difference of the topologically optimized block, solid Ti6Al4V block, and porous Ti6Al4V block in treating bone defects of total knee revision was compared by finite element analysis. The inhomogeneous femoral model was created according to the computed tomography data. Combined with porous structures, minimum compliance topology optimization subjected to the volume fraction constraint was utilized for the redesign of the metal block. The region of interest was defined as a 10 mm area of the distal femur beneath the contacting surface. The biomechanical performance of daily motions was investigated. The von Mises stress, the strain energy density of the region of interest, and the von Mises stress of metal blocks were recorded. The results were analyzed in SPSS. In terms of the region of interest, the maximum von Mises stress of the topological optimized group increased obviously, and its average stress was significantly higher than that of the other groups (*p* < 0.05). Moreover, the topologically optimized block group had the highest maximum strain energy density of the three groups, and the lowest maximum stress of block was also found in this group. In this study, the stress shielding reduction and stress transfer capability were found obviously improved through topology optimization. Therefore, the topological optimized porous block is recommended in treating bone defects of total knee revision. Meanwhile, this study also provided a novel approach for mechanical optimization in block designing.

## Introduction

The management of bone defects is a crucial issue in total knee revision (TKR). Bone defects are frequently connected with bone resorption and iatrogenic bone loss during the revision (Sheth et al. 2017). Stable reconstruction might be challenging because the management varies depending on the bone defects (Innocenti and Pianigiani 2018). Type 3 defects, according to the Anderson Orthopedic Research Institute (AORI) classification, have significant metaphyseal bone damage and cancellous bone loss (Lei et al. 2019). The most common choices are solid and porous metal augmentations (Aggarwal and Baburaj 2020). However, the current techniques have their own limitations.

Solid metal block augmentation has been applied in bone defect restoration for years. Robust structural support could be provided by the metal block (Aggarwal and Baburaj 2020). Furthermore, more host bone could be preserved due to the customizability of the metal block (Kang et al. 2019; Mozella and Cobra 2021). However, the application of metal block would generate stress shielding inevitably (Liu et al. 2021). The current metal blocks are mainly made of Ti6Al4V, which has a much higher Young’s modulus than human bone (Wang et al. 2016). Due to the large variation in material properties, stress shielding occurs and results in unbalanced loading assignments (Innocenti and Pianigiani 2018). As a result, the bone receives inadequate stress stimulation. According to Wolff’s law, the high-stress stimulation promotes the structural strengthening of the bone, whereas peri-implant bone resorption and even fracture could be produced by low-stress stimulation (Mirulla et al. 2021; Zhang et al. 2020). Hence, reducing stress shielding is necessary for the application of metal blocks.

The porous design of the metal block is an effective strategy for modifying the metal properties (Lei et al. 2021; Mozella and Cobra 2021). The equivalent Young’s modulus of the porous metal blocks could be decreased by adjusting the porosity (Arabnejad et al. 2016). This has been proven to be an effective method for narrowing the gap of material properties and reducing the stress shielding (Faizan et al. 2017). Additionally, the porous metal block has a regular porous morphology and strong connectivity which could assist in osteointegration (Lei et al. 2021; Liu et al. 2020). However, due to the absence of the ability to replicate the anisotropic and heterogeneous structures of the bone, the biomechanical property is far from optimal, and it is easy to cause local stress concentration in porosity structures, which is unfavorable for the long-term use of the metal block (Chen et al. 2020; Wang et al. 2018). Therefore, a structural redesign that could provide better stress distribution is needed for porous metal block.

Topology optimization (TO) is a structural optimization approach which could satisfy specified biomechanical standards by altering material distribution (Wu et al. 2021). Under the preset load and boundary conditions, optimal material distribution and stress shielding reduction could be achieved (Guoqing et al. 2021). Studies have concluded that the TO implant could prevent bone resorption by promoting stress transfer on bone (Alkhatib et al. 2019; Zhang et al. 2020). Liu et al. revealed that the TO porous metal block could achieve superior stress distribution than the original metal block in the treatment of uncontained tibia bone defects (Liu et al. 2021). However, there is still an absence in the biomechanical research of TO porous metal blocks in treating TKR distal femoral bone defects.

The purpose of this study was to redesign a TO porous metal block and compare the biomechanical differences in treating AORI type 3 distal femoral bone defects between the original solid metal block, porous metal block, and TO porous metal block. In this study, the TO and porous structure design of the metal block was performed, and the biomechanical behaviors under various motions were evaluated by finite element analysis (FEA).

## Material and methods

### Preparation

The volunteer is a 57-year-old male with left knee arthritis, and the computed tomography (CT) images were obtained using an Aquilion One Scanner (Toshiba, Japan) with 120 kVp, 100 mAs, B70f kernel, and 0.60 mm slice thickness. The DICOM data were imported into Mimics Medical V21 (Materialise, Belgium) to complete the reconstruction of the three-dimensional (3D) femoral model. The present study was conducted with the informed consent of the patient and the approval of the Ethics Committee of the Second Hospital of Jilin University.

### Model reconstruction

**Fig. 1:**
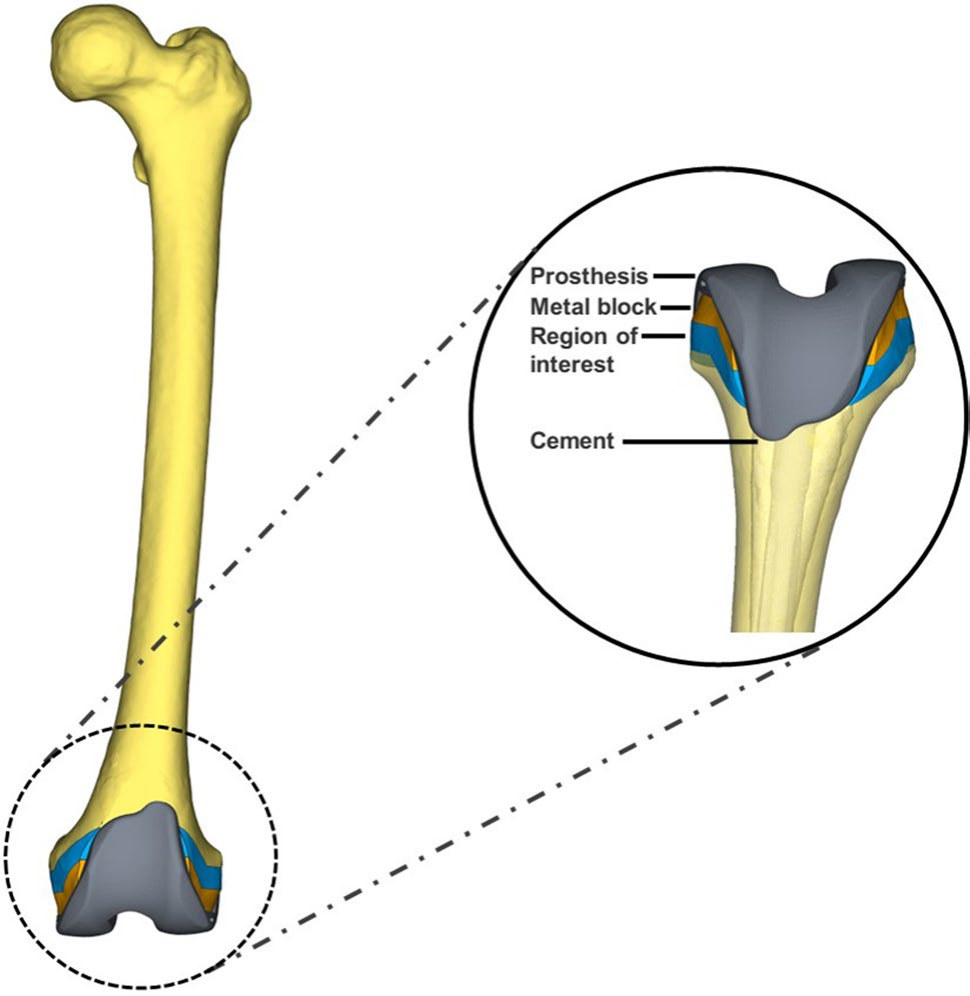
The model consists of a CCK prosthesis, metal block, region of interest, femur, and bone cement

In this study, the condylar constrained knee (CCK) prosthesis (Ikang, Beijing), which could confer varus-valgus and rotational stability, was selected for TKR (Andreani et al. 2020). The STL data of the femur and prosthesis were imported into Magics V21 (Materialise, Belgium), and the simulated operation was performed after mesh correction. The original block was obtained by the Boolean operation at the distal 5 mm plane of the femoral medial and lateral ligaments. The distal bone defects restoration of TKR was conducted in Mimics (Fig. 1). Meanwhile, the 10 mm area of the femur beneath the contacting surface of the blocks was determined as the region of interest (ROI) (Innocenti and Pianigiani 2018). In addition, the porosity and the strut thickness of the porous Ti6Al4V block were defined as 18% and 1.25 mm, respectively (Fig. 4 b), which could preserve structural stability and reduce the stress shielding (Mehboob et al. 2020). For the reservation of the geometrical characteristics, the mesh sizes of the prosthesis, metal block, and cement were defined as 1 mm, 0.8 mm, and 0.75 mm.

### Mesh convergence

The sensitivity analysis of the femur model was performed to reduce the influence of meshing differences in FEA and improve computational efficiency. The femoral models with four mesh sizes were developed for the sensitivity analysis, and the size of 0.5 mm which could preserve more geometrical features was defined as the reference group (Table 1). The element size of the femur models was 0.5 mm, 0.8 mm, 1.0 mm, and 1.2 mm, respectively. The constraint zone was defined as the region above the lesser trochanter of the proximal femur, and the load of walking was applied to the distal femoral articular surface (Table 2). The maximum von Mises stress of case 1–3 was compared with the reference group; a difference within 5% was considered as valid (Liu et al. 2021). At last, case 1 was found to be optimal since it could reduce the calculation costs while maintaining an accuracy of 98%, cases 2–3 were inaccurate (> 5%).

**Table 1:** Sensitivity analyses on mesh density for bone

Case(s)	Element size (mm)	Peak stress in bone (MPa)	Difference from the reference value (%)
Reference	0.5	78.94	–
Case 1	0.8	77.55	1.76
Case 2	1.0	74.15	6.07
Case 3	1.2	85.76	8.64

**Table 2:** Forces parameters of different motions in FEA

Motions	Force type	Force
Walking	F_M_ (N)	1125
	F_L_ (N)	750
Climbing stairs	F_M_ (N)	1160
	F_L_ (N)	773
	Medial anterior–posterior force AP_m_ (N)	−3
	Lateral anterior–posterior force AP_L_ (N)	−3
	Patella-femoral force PF (N)	567
	Internal-exteinal moment IE (Nmm)	7029
Chair-rising	F_M_ (N)	600
	F_L_ (N)	400

* F_M_, F_L_ represent the medial and the lateral force, respectively

### Material property and load setting

**Fig. 2:**
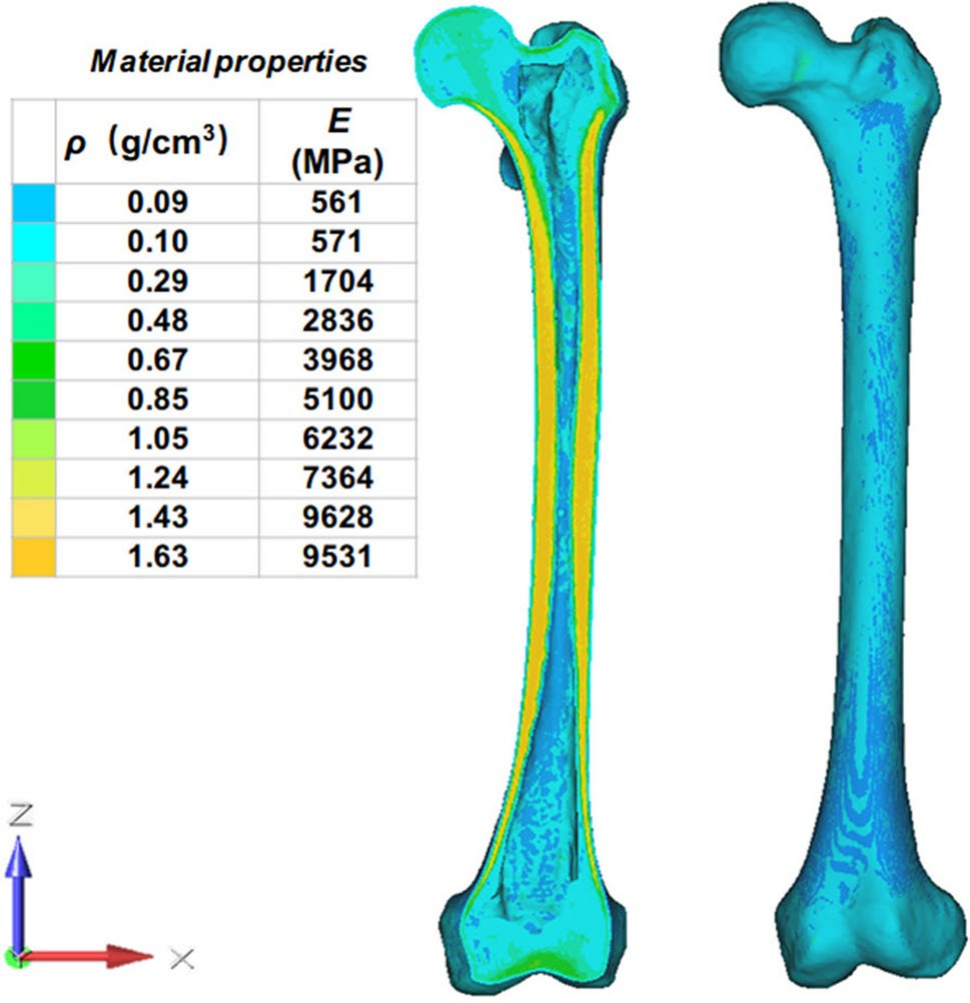
The femoral model was divided into ten material property groups with ten varied colors. ρ: Bone density; E: Elastic modulus

The material property of the TKR system (ACCK prosthesis, blocks, and bone cement) was assigned in Hypermesh (Table 3). The contacting type of Surface-to-Surface contact was defined between different components. The sticky connection which allows no sliding was set between the contacting surface of prosthesis-block, prosthesis-bone, and block-bone. The frozen contact which allows no displacement was established between the contacting surface of prosthesis-bone cement and bone-bone cement. The material property of the inhomogeneous femur model was assigned in mimics (Fig. 2). According to the empirical formulas of the material property assignment in Mimics (Mo et al. 2019; Peng et al. 2020; Zhang et al. 2020), the bone density (*ρ*) and elastic modulus (*E*) could be calculated:

1 $$\begin{array}{*{20}c} {\rho \left( {g/m^{3} } \right) = - 13.4 + 1017 \times GV\left( {HU} \right)} \\ \end{array}$$

2 $$\begin{array}{*{20}c} {E\left( {Pa} \right) = - 388.8 + 5925 \times \rho \left( {g/m^{3} } \right)} \\ \end{array}$$

**Table 3:** Material properties of the components

Component	Elasticity modulus (MPa)	Poisson’s ratio
Prosthesis	110, 000	0.33
Original solid Block	110, 000	0.33
Porous Block (18% porosity)	76, 700	0.33
TO Block (30% porosity)	53, 800	0.33
TO Block (70% porosity)	9100	0.33
Cement	3000	0.37

**Fig. 3:**
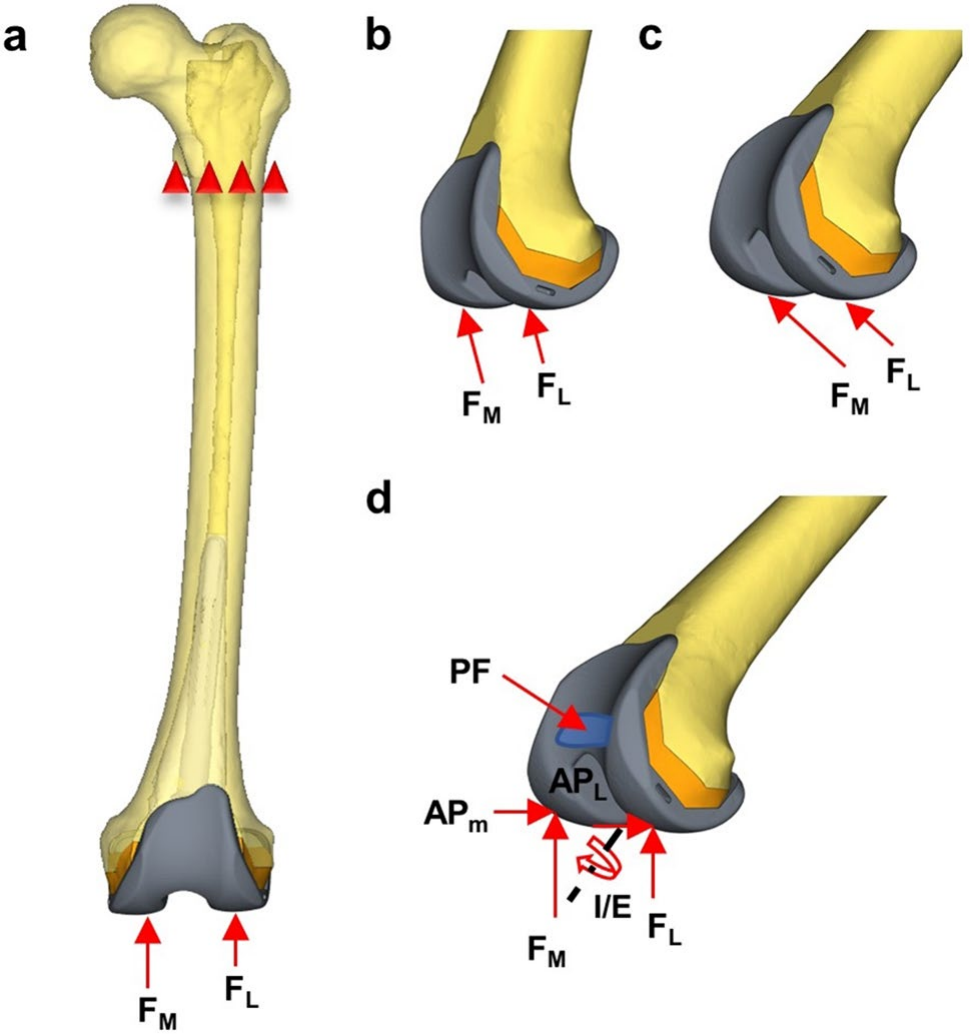
(**a**): Loads and constrains on the femur, F_M_: F_L_ = 60%: 40%. (**b**), (**c**), and (**d**) represent the forces applications of walking, chair-rising, and climbing stairs, respectively. The rbe3 regions were set in all the points of force application, such as the blue area in (**d**)

Three loading conditions of daily life were adopted for the finite element simulation (Fig. 3): 1. Walking (30° flexion of the knee): the femur is 15° flexed relative to the longitudinal axis of the human body and the load is approximately three times body weight (Innocenti and Pianigiani 2018). 2. Climbing stair (48° flexion of the knee): the maximum functional flexion angle during ascending stair motion (Conlisk et al. 2018). 3. Chair-rising (90° flexion of the knee): this motion studies the moment of rising from seated position. The magnitude of the loads is decreased compared with the other motions due to the support of the upper limbs (Burastero et al. 2020). The exact loading parameters are presented in Table 2. Based on the previous research, the force ratio of the medial and lateral condyles was defined as F_M_: F_L _= 60%: 40% (Conlisk et al. 2015). In this study, the function of “rigid bar element 3” (rbe3) in Hypermesh was utilized on the prosthesis surfaces for even force application (Liu et al. 2021). Rbe3, which selected the nodes in the contacting region, could equally distribute the force to the region by applying load to the main node. Meanwhile, all elements above the proximal lesser trochanter of the femur were constrained by six degrees of freedom (Fig. 3).

### TO and porous design

TO was conducted in the Optistruct solver of Hypermesh. The original metal block was selected as the optimized component. Under the loads and boundary conditions mentioned above, the minimum compliance of the TO region subject to the volume fraction constraint was utilized (Zhang et al. 2020). The optimization formula is as follows:

3 $$\begin{array}{*{20}c} {{\text{Objective function:}}\;\;{\text{minimize}}\left( {{\text{U}}_{{\text{c}}} } \right){;}} \\ \end{array}$$

4 $$\begin{array}{*{20}c} {Constraint:0 < \eta_{i} < 1\left( {i = 1,2,3 \ldots n} \right)} \\ \end{array}$$

5 $$\begin{array}{*{20}c} {V \le {\text{V}}_{{\text{O}}} - V*,} \\ \end{array}$$

6 $$\begin{array}{*{20}c} {V = \mathop \sum \limits_{i} \eta_{i} V_{{\text{i}}} ,} \\ \end{array}$$

7 $$\begin{array}{*{20}c} {E_{i} = E\left( {\eta_{i} } \right),} \\ \end{array}$$

8 $$\begin{array}{*{20}c} {\left\{ {\sigma_{i} } \right\} = \left[ {E_{i} } \right]\left\{ {\varepsilon_{i} } \right\},} \\ \end{array}$$

where *U*_*c*_ is the compliance, *η*_*i*_ represents the internal pseudo-density assigned to each finite element *i*, *V* is the computed volume, *V*_*0*_ is the original volume, V* represents the volume to be removed, *V*_*i*_ is the volume of element *i*, *E*_*i*_ is the elasticity modulus for each element, *E* represents the elasticity modulus, *σ*_*i*_ is the stress vector of element *i*, and *ε*_*i*_ represents the strain vector of element *i*. *η* is the density coefficient and ranges from 0 to 1. The closer the *η* value is to 0, the more material needs to be removed, and the closer the *η* value is to 1, the more original material needs to be retained.

**Fig. 4:**
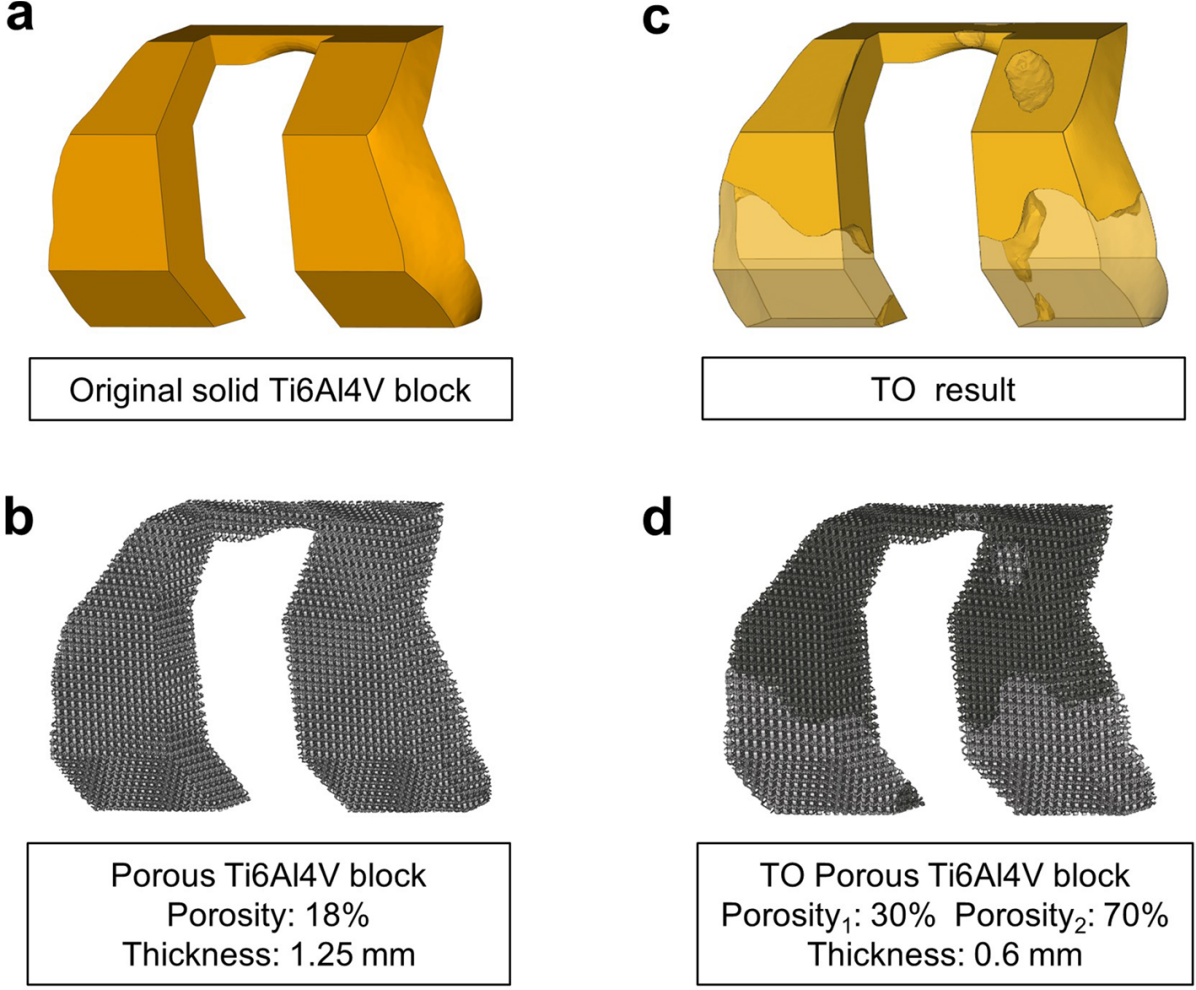
(**a**): The original Ti6Al4V block. (**b**): The porous Ti6Al4V block. (**c**): Topology optimization results, the orange part is the optimized component and the light color region is the removed component. (**d**): The topology optimization metal block designs, the dark part is the optimized component with the porosity_1_ and the gray part is the removed component with the porosity_2_

The maximum removal of the original material was set to 50% and the maximum TO iteration was defined as 20. The optimized material with a pseudo-density of 0.86 was eventually reserved to reduce the stress concentration induced by sharp geometrical features (Fig. 4 c). Meanwhile, the post-processing and the extraction of the TO design were conducted in Hyperview (Altair Engineering, USA). The porous structures were designed in Magics. The body-centered cubic unit which has a reliable mechanical property was selected for the fundamental unit (Guoqing et al. 2021). The optimized part was built with a 30% porosity and strut thickness of 0.6 mm to ensure structural stability, while the 70% porosity and the strut thickness of 0.6 mm were assigned to the removed part for osteointegration (Fig. 4 d) (Arabnejad et al. 2016; Liu et al. 2021). Rather than using the porous structures directly, the equivalent modulus of the porous structures was utilized in this study. Previous studies have verified the correlation between porosity and material property, and the equivalent elastic modulus of porous structures could be calculated (Alkhatib et al. 2019).

### Finite element analysis

The FEA was conducted by Optistruct solver in Hypermesh, the stress distribution and the strain energy density (SED) were mainly studied. The results were analyzed by single factor analysis of variance in SPSS V21.0 software (IBM, United States), and *p* < 0.05 was considered significant.

## Results

### Topology optimization design

The TO result is illustrated in Fig. 4. The TO design experienced 4 iterations and the 49% volume of the original block was removed. The removed portions were mainly concentrated on the posterior of both condyles. The optimized areas were designed with a porosity of 30%, whereas the removed portions had a porosity of 70% (Fig. 4 d).

### Maximum von Mises stress of the ROIs

**Fig. 5:**
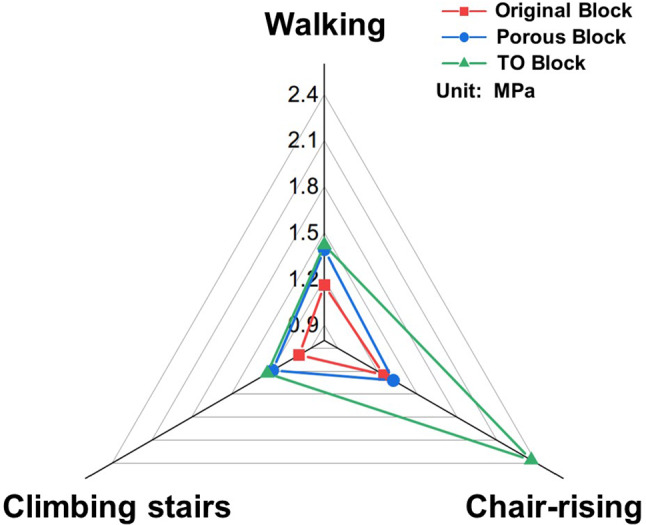
The maximum von Mises stress of the ROIs under the motion of walking, climbing stairs, and chair-rising

The maximum von Mises stress of ROIs is shown in Fig. 5. In the walking state, the maximum stress of ROIs in the original metal block, porous metal block, and the TO block was 1.16 MPa, 1.39 MPa, and 1.42 MPa, respectively. In the chair-rising state, the ROIs maximum stress of the original metal block, porous metal block, and the TO block group was 1.25 MPa, 1.32 MPa, and 2.36 MPa, and that of the climbing stair state was 0.99 MPa, 1.19 MPa, and 1.23 MPa.

### Average von Mises stress of the ROIs

The average von Mises stress of the ROIs under different motions is presented in Table 4. The average stress of the ROI in the TO block was significantly higher than that of the other groups in all the studied cases (*p* < 0.05).

**Table 4:** Average von Mises stress (MPa) of ROIs

Group	Walking	Climbing stairs	Chair-rising
	Mean ± SD (MPa)	Mean ± SD (MPa)	Mean ± SD (MPa)
Original block	0.08 ± 0.02	0.08 ± 0.04	0.06 ± 0.02
Porous block	0.10 ± 0.03	0.09 ± 0.04	0.07 ± 0.03
TO block	0.13 ± 0.03	0.11 ± 0.03	0.07 ± 0.05

### The SED of the ROIs

**Fig. 6:**
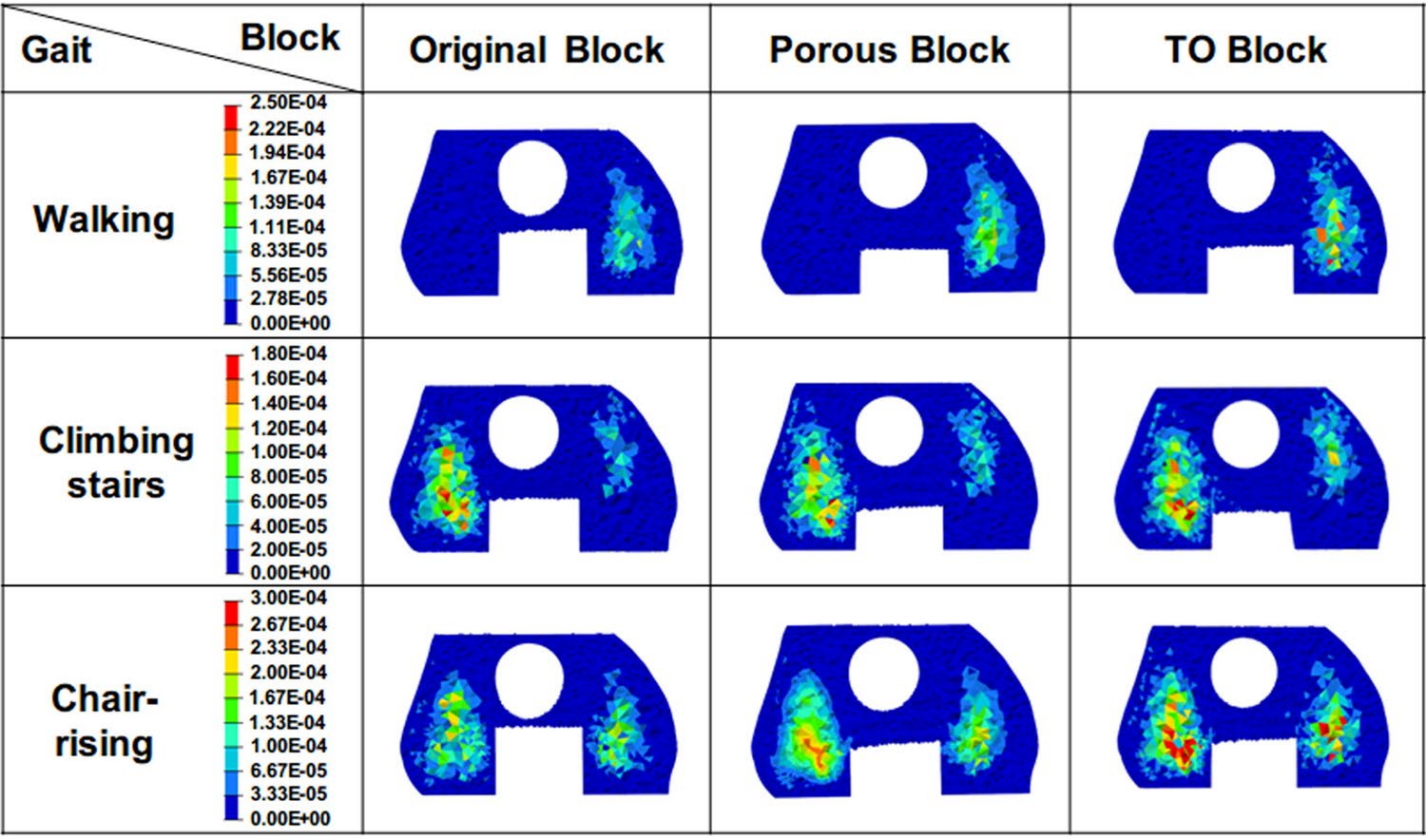
The strain energy density of ROIs in the original metal block, porous metal block, and the TO block under the conditions of walking, climbing stairs, and chair-rising

The SED of the ROIs in various motions is shown in Fig. 6. In the walking state, the maximum SED of the original metal block, porous metal block, and the TO block was 190 Pa, 200 Pa, and 270 Pa, respectively, and in the climbing stairs state, it was 130 Pa, 140 Pa, and 170 Pa. In the chair-rising condition, the maximum SED of the original metal block, porous metal block, and TO block was 210 Pa, 300 Pa, and 350 Pa, respectively.

### Von Mises stress of metal augmentations

**Fig. 7:**
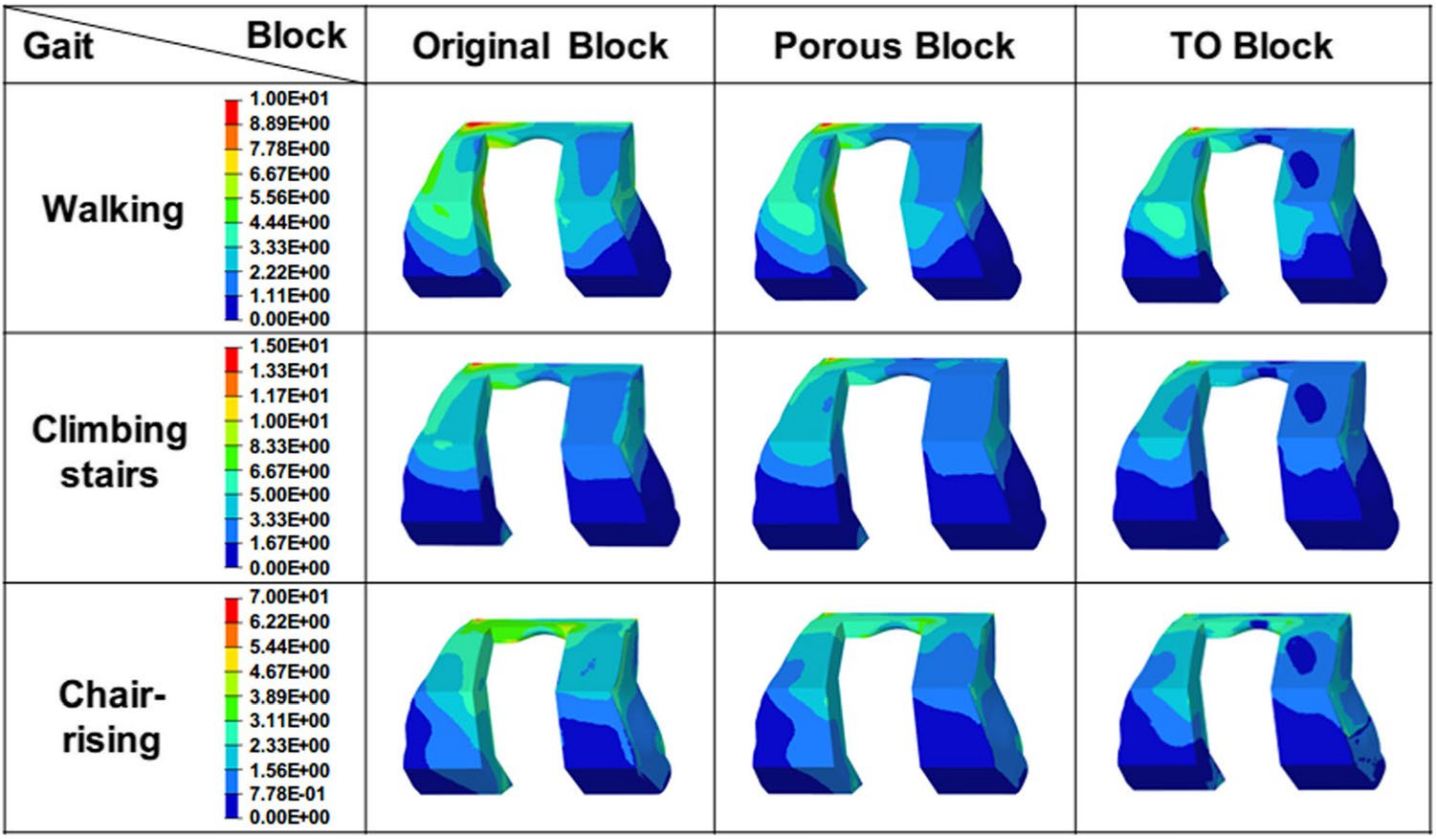
The von Mises stress distribution of the original metal block, porous metal block, and the TO block under the conditions of walking, climbing stairs, and chair-rising

The von Mises stress distribution of the original metal block, porous metal block, and the TO block under different motions is presented in Fig. 7. In the walking state, the maximum von Mises stress of the original metal block, porous metal block, and the TO block was 17.68 MPa, 15.3 MPa, and 11.73 MPa, respectively. Whereas in the climbing stairs state it was 19.5 MPa, 14.98 MPa, and 13.15 MPa. The maximum von Mises stress of the original metal block, porous metal block, and the TO block in the chair-rising condition was 8.9 MPa, 7.43 MPa, and 6.62 MPa, respectively. The high-stress regions of the three metal augmentations were mainly concentrated on the anterior portion of the blocks, with the TO block having the fewest high-stress regions in all motions studied.

## Discussion

The management of bone defects is a challenging area in TKR. It is generally accepted that metal block augmentation could provide dependable structural support for the restoration of bone defects (Innocenti et al. 2014; Innocenti and Pianigiani 2018). However, the stress shielding and osteointegration of metal block augmentations are crucial concerns for structure restoration (Aggarwal and Baburaj 2020; Kornah et al. 2019). The current study created a TO grid-graded porous metal block and compared its biomechanical performance to that of a solid metal block and a porous metal block in treating TKR distal femoral bone defects.

Structural redesign of the implant was generally considered an effective approach for avoiding stress shielding and bone resorption. Different from material alteration, structural redesign saves time and costs relatively (Zhang et al. 2020). Furthermore, the diversity of design makes it possible to achieve superior biomechanical performance (Wu et al. 2021). Al-Tamimi et al. demonstrated that reliable mechanical support and stress shielding reduction could be achieved by the new TO plate (Al-Tamimi et al. 2017). In the treatment of tibia bone defects, Liu et al. concluded that the TO metal augmentation combined with graded porous structures could lower the equivalent elastic modulus and promote osteointegration (Liu et al. 2021). According to the previous studies, the TO porous block was selected for its rational material distribution and superior biomechanical performance. In this study, the biomechanical differences in treating distal femoral bone defects were evaluated.

FEA, which serves as the foundation for TO, is an effective tool for biomechanical evaluation. The authenticity of the reconstructed model and the validity of the loading conditions are essential for the accuracy of the analysis. The femoral model of this study, which was reconstructed based on the CT images, is beneficial for the biomechanical evaluation of varied bone densities and could effectively ensure the reliability of TO design (Zhang et al. 2020). The motions of walking, chair-rising, and climbing stairs were also included in the simulation analysis. The studied motions are the main contents of postoperative rehabilitation training (Chen et al. 2021; Conlisk et al. 2018), the adoption of these motions will help to improve the simulation degree of the biomechanical study. Moreover, several biomechanical indicators were involved in this study for the objective analysis, including the von Mises stress, the SED of ROI, and the stress distribution of blocks.

The evaluation of the von Mises stress in ROI was considered an effective indicator which could accurately reflect the stress transfer capacity and stress shielding (Innocenti and Pianigiani 2018). Higher peak stress and average stress of ROI indicate a better stress transfer capacity of the implants and less stress shielding. In the present study, the peak stress of ROI in the TO block group was obviously higher than that of the solid metal block group (up to 47%) and the porous metal block (up to 18%), which showed that more stress was transferred to the ROI in the TO block group, indicating better stress transfer capacity of the TO block. What’s more, the highest average stress of ROI was also found in the TO block group by statistical analysis (*p* < 0.05), implying more high-stress stimulation on the contacting regions. The presence of these stimulations indicated that the application of TO block could help to promote stress transfer and osteointegration, which is crucial for the prevention of bone resorption (Castillo and Leucht 2015; Cheong et al. 2020).

The SED is another index for the evaluation of stress shielding and the prediction of bone resorption (Mathai et al. 2021; Nag and Chanda 2021). A higher bone SED indicates more mechanical stimulation, lower stress shielding, and less bone resorption (Zhang et al. 2020). In all the studied cases, the peak SED of the ROI in the TO block group improved greatly compared to the original metal block group (up to 67%) and the porous metal block group (up to 35%), which suggested more mechanical energy was received on the bone of the TO group, indicating the stress transferring capability of the TO block was superior. Besides, the higher SED of the TO block appeared more in the high energy exercises, indicating that the stress transfer capability would be enhanced in these motions. Compared to the other block groups, the high-SED area of the TO block group was found to increase obviously, suggesting more mechanical stimulation and better osteointegration.

To evaluate the stress transfer capability clearly, the stress distribution of the various blocks was also studied. The lower stress and less high-stress area of the block indicate better stress transfer capability (Peng et al. 2021; Vogel et al. 2021). In all studied motions, the peak stress of the TO block was found lower than that of the original block (up to 34%) and the porous block (up to 11%), and the distribution of high stress in the TO block was the least. Meanwhile, the stress alteration of the TO block under various motions was found more stable than the others, indicating that the stress transfer capability of the TO block was more reliable. The results showed that better stress assignment of different components could be achieved by the TO block, which means the stress transfer capability of the TO block is superior, indicating that the rational material distribution and the lower equivalent elastic modulus enhanced the stress transfer capacity effectively.

The above findings revealed that superior biomechanical performance could be achieved by the porous TO block. Compared with the other block designs, the lower equivalent elastic modulus and the TO design effectively improved the stress transfer capability and decreased the stress shielding while maintaining reliable structural support. More spaces for bone ingrowth were preserved in the TO block, which suggested the superiority of osteointegration ability in the TO block. Under the mechanical stimulations, the osteointegration of the TO block would help to improve the long-term stability (Cheong et al. 2020).

Most notably, this is the first study to our knowledge to assess the biomechanical performance of different metal block designs in treating TKR distal femoral bone defects. In restoring distal femoral bone defects, the current study expands on earlier studies by confirming that TO block design could provide superior biomechanical performance. (Innocenti and Pianigiani 2018; Liu et al. 2021). Zhang et al. used topology optimization to create a porous proximal tibial metal block that outperformed the solid block biomechanically (Zhang et al. 2020). In their study, the maximum stem stress in the TO group was 39.1% lower than in the solid block group, while the tibial maximum von Mises stress was 39.6% higher and the maximum SED was 61.5% higher. In the current study, an ROI was proposed to more specifically measure changes in biomechanical performance. When compared to the standard solid block, the maximum stress of the TO group ROI increased by 47%, the maximum SED of the ROI increased by 67%, and the maximum stress of the TO group block decreased by 34%. This suggests that the TO design of this study is more in line with biomechanical requirements. Our results provide compelling biomechanical evidence for the treatment of distal femoral bone defects in TKR and suggest that this approach appears to be effective in the biomechanical improvement and bone resorption prevention.

The current study still has some limitations which need to be improved in the future. Firstly, despite the fact that several mechanical indicators were adopted for the biomechanical evaluation, the manufacturing and mechanical test of blocks are still needed for verification in the future. Secondly, the TO block design in this study was based on the specific patient, which would be constrained in clinical practice. For the convenience of clinical application, a large sample size will be considered in designing process in the future to improve universality.

## Conclusions

The focus of this study was to improve the biomechanical performance of the metal block augmentation using TO porous designs. The biomechanical difference was evaluated by FEA. Compared with the other blocks, the stress and the SED of the bone in the TO group increased greatly, and the stress of the TO block decreased clearly. The novel TO block may effectively promote stress transfer while avoiding stress shielding and bone resorption.
